# Plan Quality Analysis of Automated Treatment Planning Workflow With Commercial Auto-Segmentation Tools and Clinical Knowledge-Based Planning Models for Prostate Cancer

**DOI:** 10.7759/cureus.41260

**Published:** 2023-07-01

**Authors:** Jacob Adams, Kirk Luca, Xiaofeng Yang, Pretesh Patel, Ashesh Jani, Justin Roper, Jiahan Zhang

**Affiliations:** 1 Department of Radiation Oncology, Emory University, Atlanta, USA

**Keywords:** workflow validation, knowledge-based planning, automated treatment planning, plan quality, auto-segmentation, prostate cancer

## Abstract

This study evaluated the feasibility of using artificial intelligence (AI) segmentation software for volume-modulated arc therapy (VMAT) prostate planning in conjunction with knowledge-based planning to facilitate a fully automated workflow. Two commercially available AI software programs, Radformation AutoContour (Radformation, New York, NY) and Siemens AI-Rad Companion (Siemens Healthineers, Malvern, PA) were used to auto-segment the rectum, bladder, femoral heads, and bowel bag on 30 retrospective clinical cases (10 intact prostate, 10 prostate bed, and 10 prostate and lymph node). Physician-segmented target volumes were transferred to AI structure sets. In-house RapidPlan models were used to generate plans using the original, physician-segmented structure sets as well as Radformation and Siemens AI-generated structure sets. Thus, there were three plans for each of the 30 cases, totaling 90 plans. Following RapidPlan optimization, planning target volume (PTV) coverage was set to 95%. Then, the plans optimized using AI structures were recalculated on the physician structure set with fixed monitor units. In this way, physician contours were used as the gold standard for identifying any clinically relevant differences in dose distributions. One-way analysis of variation (ANOVA) was used for statistical analysis. No statistically significant differences were observed across the three sets of plans for intact prostate, prostate bed, or prostate and lymph nodes. The results indicate that an automated volumetric modulated arc therapy (VMAT) prostate planning workflow can consistently achieve high plan quality. However, our results also show that small but consistent differences in contouring preferences may lead to subtle differences in planning results. Therefore, the clinical implementation of auto-contouring should be carefully validated.

## Introduction

Prostate cancer (PCa) patients represent a significant percentage of the total patient population treated in radiotherapy clinics worldwide. PCa is the most frequently diagnosed cancer in men, with 1.4 million new cases diagnosed per year, leading to 375,000 deaths worldwide [1]. With the increasing incidence rate of PCa due to an aging male population combined with an increase in the overall therapeutic utilization of radiation therapy for all treatment sites, the cumulative number of cancer patients has been projected to significantly increase [1]. This cumulative increase has been projected to create an industry-wide surge in the demand for treatment planning staff and resources: a previous study has predicted a shortage of medical dosimetrists beginning in 2021 and extending into 2030 [2]. In response to these shortages, clinical workloads are estimated to increase, which poses challenges to clinical planning efficiency and quality. In general, it is important to provide timely access to radiotherapy, as extended delays correlate with an increased risk of local recurrence [3] and poor survival rates [4]. At the same time, ensuring high plan quality is also critical, as suboptimal quality has been linked to poor clinical outcomes [5]. The demand to provide a greater number of high-quality treatment plans is a real challenge due to a shortage of qualified medical dosimetrists.

In the current treatment planning workflow, image segmentation and plan optimization are two bottlenecks that consume tremendous time and energy from a medical dosimetrist, yet at the same time present opportunities for improved efficiency through innovation. Segmenting organs at risk (OARs), during the treatment planning process, serves as a crucial and necessary step to accurately delineate radiosensitive healthy tissues. Traditionally, segmentation has been performed manually, which requires significant time and effort by the planner to draw OAR boundaries slice by slice on a CT scan. Though a number of segmentation tools have been clinically available for over a decade, dosimetrists have been tasked with the time-consuming manual edits. Thanks to recent breakthroughs, deep learning-based auto-segmentation algorithms have achieved substantial improvements over the previous generation of atlas-based algorithms [6]. It has been shown that commercial software programs can generate contours comparable to manual contours, with variations reduced to those seen at inter-human-observer levels [7]. Another time-consuming yet crucial part of the planning workflow is setting plan optimization, which requires strategic plan constraints. The dosimetrist is typically tasked with setting these constraints through a manual trial-and-error process. The resulting plan quality depends strongly on planner skills [8]. Knowledge-based planning (KBP) methods have been developed to guide this inverse planning process based on empirical data from prior high-quality plans [9]. It has been shown that KBP can effectively reduce intra- and inter-planner plan quality variations and increase planning efficiency [10].

In light of the significant staffing challenges and the increased demands for high-quality treatment plans, the goal of this study is to evaluate the feasibility and quality of an automated workflow for VMAT prostate treatment planning facilitated by artificial intelligence (AI) auto-segmentation and KBP.

## Materials and methods

Male pelvic anatomy was segmented on CT image sets acquired from 30 clinical prostate patients. Among these 30 cases, there were 10 intact prostate cases, 10 prostate and lymph node cases, and 10 prostate bed cases. Three sets of contours were generated for each case: manual physician contours, Radformation AutoContours (Radformation, New York, NY), and contours auto-segmented by Siemens AI-Rad Companion (Siemens Healthineers, Malvern, PA), which is built into Syngo.via (Siemens Healthineers). We shall refer to these three datasets as MD, Radformation, and Syngo.via in the following sections of this paper. All manually drawn contours were generated and reviewed by staff physicians at a university hospital. The Radformation and Syngo.via artificial intelligence (AI) software contoured the bowel bag, femoral heads, bladder, and rectum. MD target volumes were transferred to these structure sets. VMAT treatment plans were created for each contour set using an in-house RapidPlan model, which was created specifically for intact prostate, prostate bed, or prostate and lymph nodes. Varian Eclipse photon optimizer (PO; Varian Medical Systems, Palo Alto, California) V16.1 was used for plan optimization, and the anisotropic analytical algorithm (AAA) V16.1 was used for the final dose calculations. Following the dose calculation, plans were normalized so that the prescription dose covered 95% of a PTV. After normalization, plans optimized using AI contours were reassigned to the MD structure set. These plans were then recalculated using prefixed monitor units. By taking this approach, AI contours were used for plan optimization, but the dose-volume histogram (DVH) analysis was based on the gold standard MD contours.

Clinically relevant plan quality metrics, including bladder V40Gy (%), bladder V70Gy (cc), rectum V45Gy (%), rectum V70Gy (cc), and femoral head Dmean (Gy), were evaluated for all plans. A one-way analysis of variation (ANOVA) test was used to evaluate the statistical significance of the differences in plan metrics between plan groups (MD, Radformation, Syngo.via). In this work, we have selected alpha=0.05/6=0.0083 as the significance threshold, with Bonferroni correction applied to six tests. Any result smaller than 0.0083 indicates statistical significance within the group.

## Results

One-way ANOVA

The null hypothesis for the one-way ANOVA tests is that all matching samples come from the same distribution. Based on the one-way ANOVA results shown in Table 1, no significant difference was observed between the three sets of plans for intact prostate, prostate bed, or prostate and lymph node.

**Table 1:** One-way analysis of variance (ANOVA) test between the three sets of plans (MD, Radformation, and Syngo.via)

	Intact prostate	Prostate bed	Prostate lymph node
Bladder V_40Gy_ (%)	0.882	0.659	0.748
Bladder V_70Gy_ (cc)	0.231	0.467	0.669
Rectum V_45Gy_ (%)	0.036	0.023	0.031
Rectum V_70Gy_ (cc)	0.027	0.081	0.323
Left femoral head D_mean_ (Gy)	0.581	0.071	0.031
Right femoral head D_mean_ (Gy)	0.973	0.138	0.274

Intact prostate

**Figure 1:**
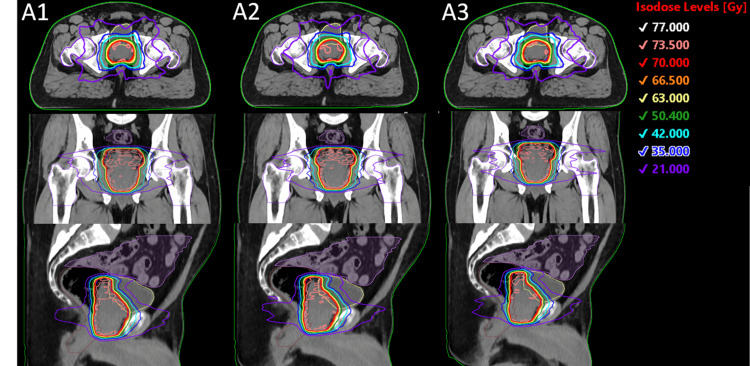
Dose distributions of plans created with (A1) MD contours, (A2) Radformation auto-contours, and (A3) Syngo.via auto-contours

**Figure 2:**
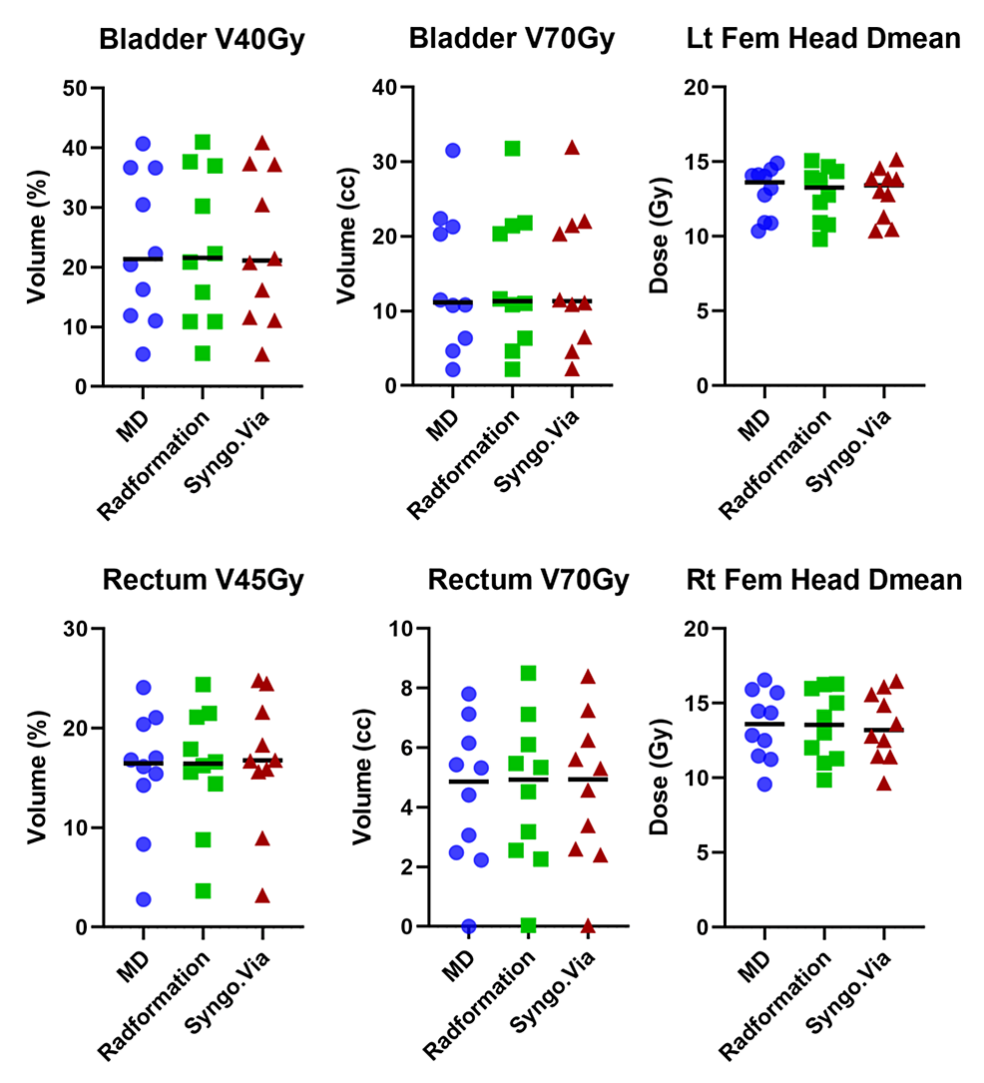
Dosimetric results for intact prostate patients

Figure 1 shows the dose distribution of all three plans for a randomly selected intact prostate patient. It is evident that the contours and final dose distributions are consistent between all three plans. This can be confirmed by Figure 2, which shows the dosimetric results of all intact prostate plans included in this study.

Table 2 lists the averages and standard deviations of dosimetric values for the three sets of intact prostate plans. The most notable difference is between the rectum V45Gy of Syngo.via (16.6 % ± 6.3 %) and MD plans (15.6 % ± 5.9 %). However, the difference is not statistically significant.

**Table 2:** Dosimetric values for intact prostate plans

	MD	Radformation	Syngo.Via
Bladder V_40Gy_ (%)	23.2±11.7	23.2±12.0	23.2±11.9
Bladder V_70Gy_ (cc)	14.2±8.8	14.2±8.9	14.3±8.9
Rectum V_45Gy_ (%)	15.6±5.9	16.0±5.8	16.6±6.3
Rectum V_70Gy_ (cc)	4.4±2.3	4.5±2.4	4.6±2.4
Left femoral head D_mean_ (Gy)	13.0±1.6	12.8±1.7	12.9±1.6
Right femoral head D_mean_ (Gy)	13.5±2.2	13.5±2.3	13.5±2.2

Prostate bed

**Figure 3:**
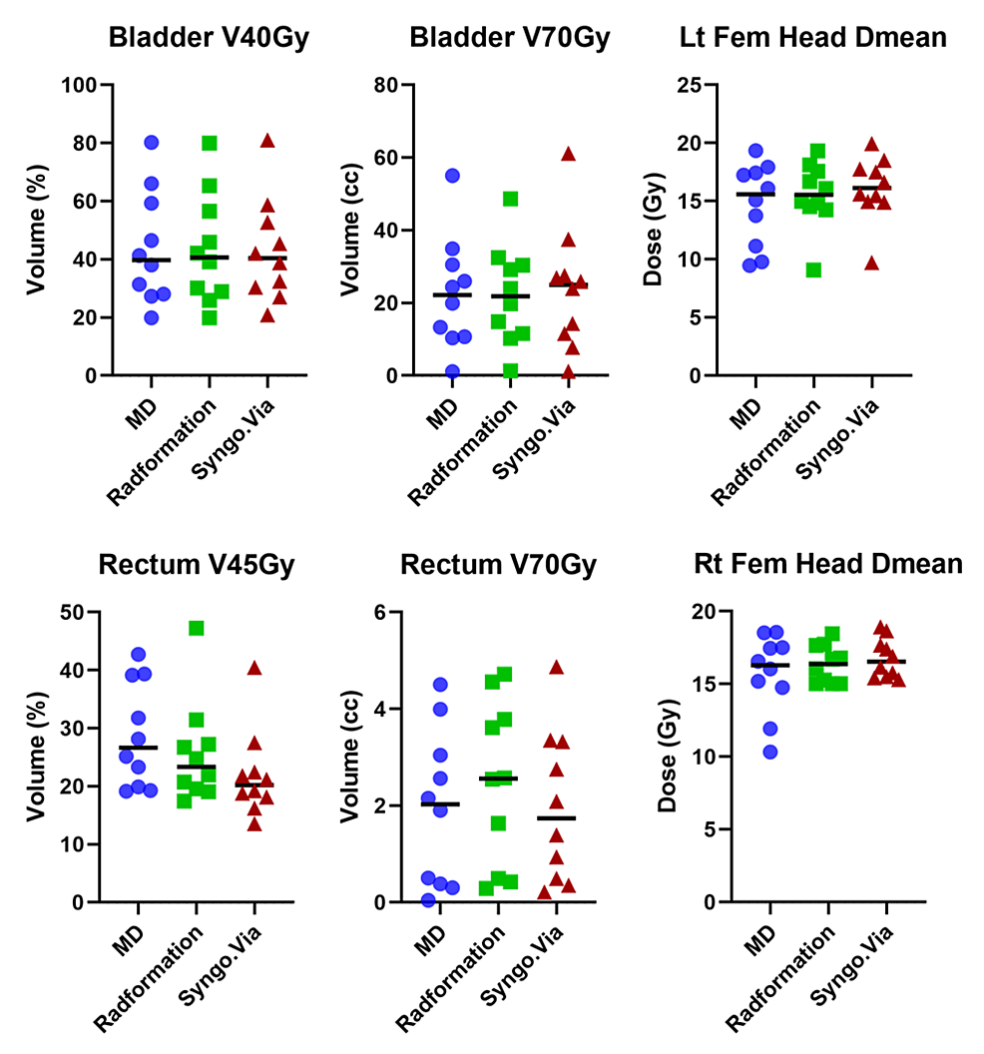
Dosimetric results for prostate bed patients

Table 3 and Figure 3 show the dosimetric values for the three sets of prostate bed plans. The largest dose difference for all contour sets was the rectum. Syngo.via scored the lowest rectal D45Gy average dose of 21.9 cc in comparison to the highest rectal D45Gy average dose of 28.9 Gy in the MD contouring set. The tradeoff seems to be the bladder V70Gy: MD plans achieved 21.6 cc ± 27.1 cc while the Syngo.via plans only achieved 23.5 cc ± 20.7 cc. The lowest prostate bed femoral head dose was recorded for the MD contour set at 15.2 Gy in comparison to Syngo.via, which had the highest Dmean average femoral dose of 16.4 Gy.

**Table 3:** Dosimetric values for prostate bed plans

	MD	Radformation	Syngo.Via
Bladder V_40Gy_ (%)	43.8±18.3	43.4±18.0	42.9±16.8
Bladder V_70Gy_ (cc)	22.6±14.6	22.2±12.9	23.8±16.2
Rectum V_45Gy_ (%)	28.8±8.6	25.6±8.3	21.9±7.1
Rectum V_70Gy_ (cc)	1.9±1.5	2.5±1.6	2.0±1.5
Left femoral head D_mean_ (Gy)	14.7±3.4	15.6±2.7	16.1±2.6
Right femoral head D_mean_ (Gy)	15.7±2.6	16.4±1.2	16.8±1.3

**Figure 4:**
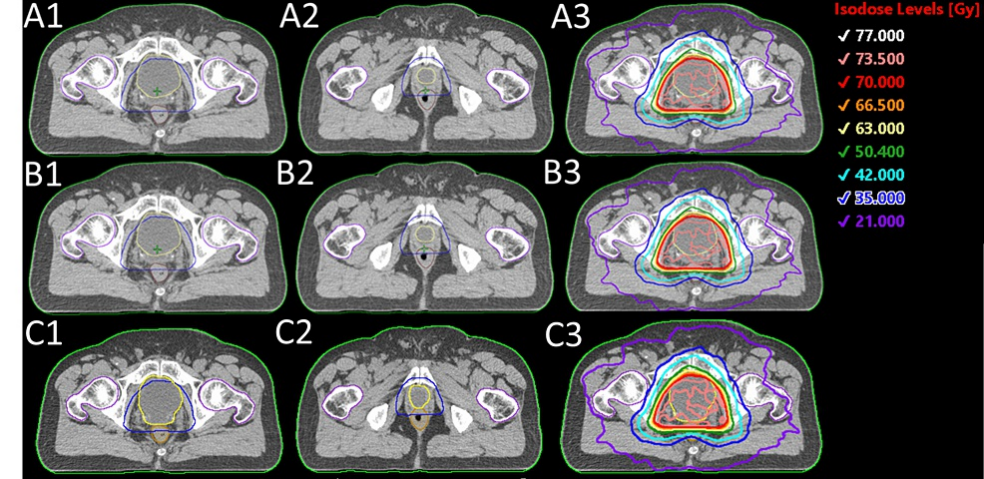
Transaxial views of prostate bed contours and plans created with (A1-A3) manual contours reviewed by physicians, (B1-B3) Radformation auto-segmentation, (C1-C3) Syngo.via auto-segmentation

Figure 4 shows an example case that could help explain the discrepancy between these plans. While MD and Radformation contours match very well. It is evident that the Syngo.via algorithm contoured additional OAR volumes inside the PTV for both the bladder and the rectum and thereby resulted in greater penalties on these OARs during plan optimization.

Prostate and lymph node

**Figure 5:**
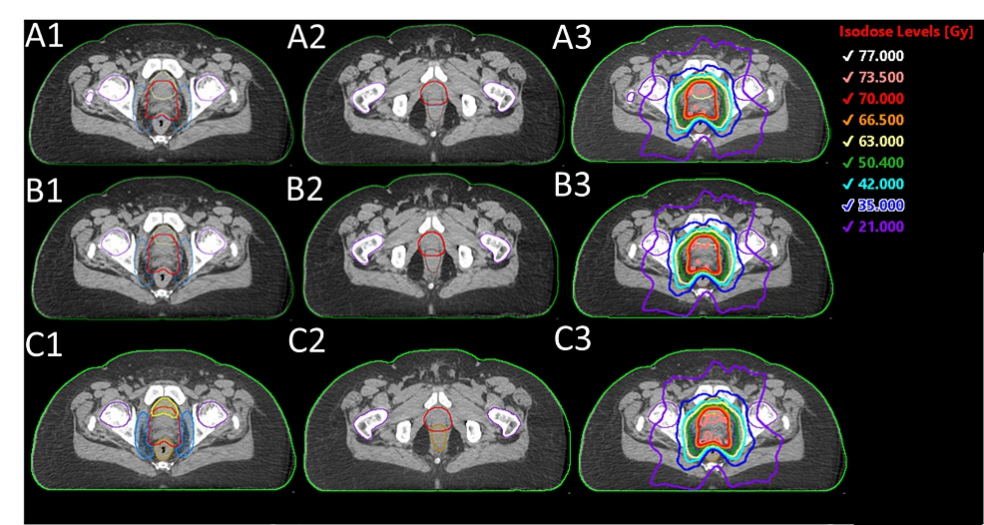
Transaxial views of prostate and lymph node contours and plans created with (A1-A3) manual contours reviewed by physicians, (B1-B3) Radformation auto-segmentation, and (C1-C3) Syngo.via auto-segmentation

Figure 5 shows a representative prostate and lymph node case. The contours created by Radformation and Syngo.via are visually comparable with the clinical contours. The final dose distributions are also highly similar between the three plans.

**Figure 6:**
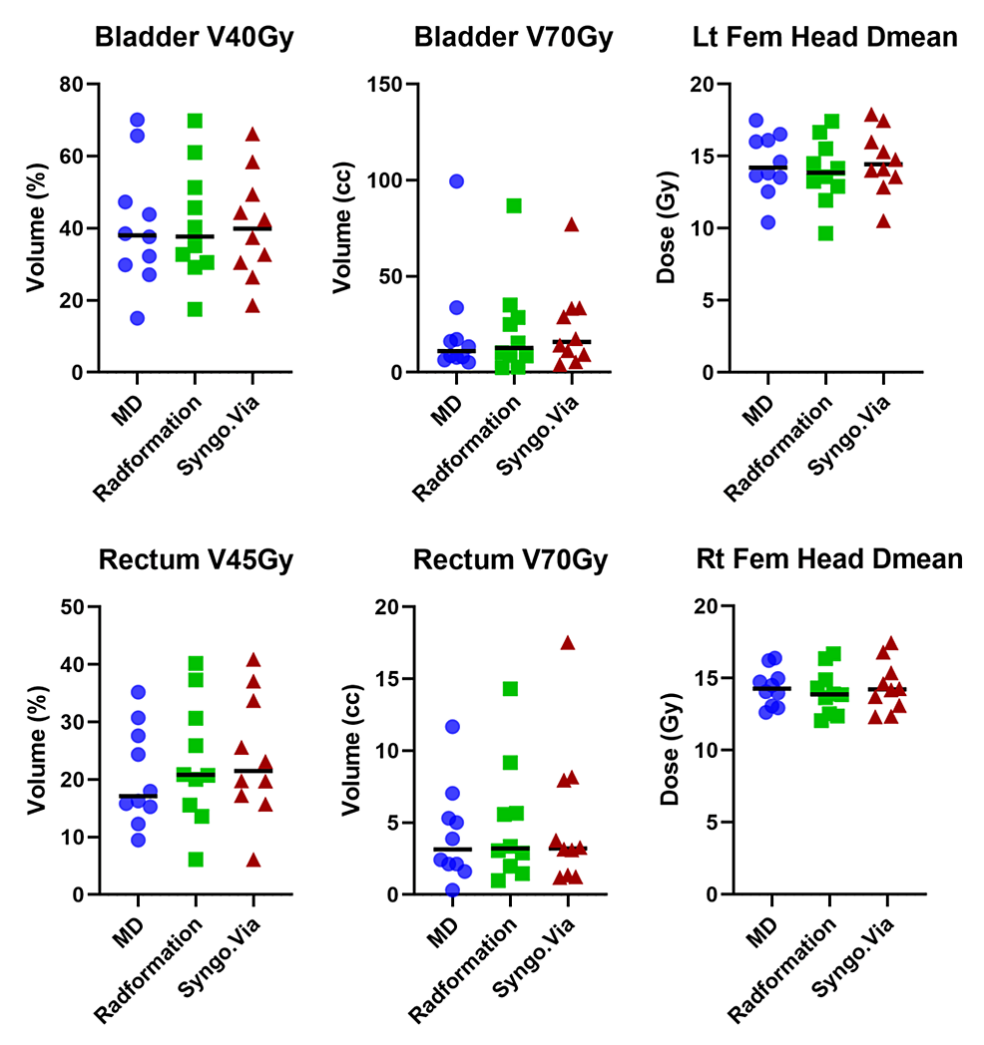
Dosimetric results for prostate and lymph node patients

Table 4 and Figure 6 show the dosimetric values for the three sets of prostate and lymph node plans. The largest dose difference for all contour sets was the rectum. The MD contouring set scored the lowest rectal D45Gy average dose of 20.5 cc in comparison to the highest rectal D45Gy average dose of 23.9 cc in the Syngo.via contouring set. Syngo.via exhibited the highest left femoral head Dmean of 14.7 Gy compared to the lowest left femoral head Dmean of 14.0 Gy of Radformation.

**Table 4:** Dosimetric values for prostate and lymph node plans

	MD	Radformation	Syngo.Via
Bladder V_40Gy_ (%)	40.7±16.1	41.3±15.1	40.7±13.9
Bladder V_70Gy_ (cc)	21.6±27.1	22.3±23.9	23.5±20.7
Rectum V_45Gy_ (%)	20.5±8.0	23.1±10.1	23.9±10.1
Rectum V_70Gy_ (cc)	4.1±3.2	4.8±3.9	5.1±4.8
Left femoral head D_mean_ (Gy)	14.5±2.0	14.0±2.2	14.7±2.1
Right femoral head D_mean_ (Gy)	14.4±1.2	14.1±1.5	14.4±1.6

## Discussion

Accurate contouring of the OARs is an essential requirement for crafting treatment plans that achieve sufficient target coverage while adequately sparing the healthy tissues to reduce the risk of normal tissue toxicities. In this study, no substantial contour errors were observed, and no contour edits were required for any contoured structures. The ability to generate accurate contours without needing edits reduces the human time required for treatment planning and potentially shortens the time between simulation and treatment so that a patient has faster access to radiotherapy.

Note that both AI auto-contouring software programs allow the planner to review and edit auto-segmented contours prior to export to the treatment planning system. This function was not utilized in the testing, as the study aimed to determine clinical acceptability based on an automatic workflow. This provided a significant decrease in planner-dependent cognitive input and time investment, allowing for every patient included in the study to be contoured and planned within a day. Even with these encouraging results, careful contour and plan review is strongly recommended in clinical practice. Yet even with this review, results from this study suggest that a high percentage of plans could be clinically acceptable without any human input during contouring or plan optimization, thereby improving efficiency without compromising on quality.

Manual contouring for prostate radiotherapy is labor-intensive and time-consuming. For clinics in low socio-economic areas that experience high patient load but suffer from staffing challenges due to limited resources, patients may face extended wait time between simulation and treatments. Moderate to high-risk patients may face an undue risk if their treatments are delayed3,4. In this study, we demonstrated that no statistically significant difference was observed from the plans created based on the radiation oncologist’s manual contours versus Radformation or Syngo.Via auto-contours. The fully automated workflow with auto-segmentation and knowledge-based planning has the potential to address the planning efficiency bottleneck and reduce treatment latency.

One limitation of this work is the lack of direct comparisons between manual contours and commercial software contours in terms of segmentation metrics such as the Dice similarity score and mean Hausdorff distance. However, there are a growing number of papers that benchmark the contouring accuracy of commercial auto-segmentation algorithms [11,12]. It is expected that the contouring accuracy of this study is similar to previous studies on this topic. Another potential factor that has not been taken into account in this study is the differing contouring preferences among physicians. This could lead to increased contouring inaccuracy. Both of these limitations warrant further studies on this topic.

## Conclusions

This study has evaluated plans generated by an automated workflow powered by commercial auto-segmentation and KBP software. The results indicate that auto-segmented contours can be directly used to generate plans with KBP without sacrificing plan quality. However, small but consistent differences in contouring preferences may lead to subtle differences in planning results. Therefore, the clinical implementation of auto-contouring still needs to be carefully validated. 

## References

[1] Wang L, Lu B, He M, Wang Y, Wang Z, Du L (2022). Prostate cancer incidence and mortality: global status and temporal trends in 89 countries from 2000 to 2019. Front Public Health.

[2] Pan HY, Haffty BG, Falit BP, Buchholz TA, Wilson LD, Hahn SM, Smith BD (2016). Supply and demand for radiation oncology in the United States: updated projections for 2015 to 2025. Int J Radiat Oncol Biol Phys.

[3] Huang J, Barbera L, Brouwers M, Browman G, Mackillop WJ (2003). Does delay in starting treatment affect the outcomes of radiotherapy? A systematic review. J Clin Oncol.

[4] E C, Dahrouge S, Samant R, Mirzaei A, Price J (2005). Radical radiotherapy for cervix cancer: the effect of waiting time on outcome. Int J Radiat Oncol Biol Phys.

[5] Peters LJ, O'Sullivan B, Giralt J (2010). Critical impact of radiotherapy protocol compliance and quality in the treatment of advanced head and neck cancer: results from TROG 02.02. J Clin Oncol.

[6] Gibbons E, Hoffmann M, Westhuyzen J, Hodgson A, Chick B, Last A (2023). Clinical evaluation of deep learning and atlas-based auto-segmentation for critical organs at risk in radiation therapy. J Med Radiat Sci.

[7] Wong J, Fong A, McVicar N (2020). Comparing deep learning-based auto-segmentation of organs at risk and clinical target volumes to expert inter-observer variability in radiotherapy planning. Radiother Oncol.

[8] Nelms BE, Robinson G, Markham J (2012). Variation in external beam treatment plan quality: an inter-institutional study of planners and planning systems. Pract Radiat Oncol.

[9] Zhang J, Ge Y, Wu QJ (2022). Knowledge-based treatment planning. Machine and Deep Learning in Oncology, Medical Physics and Radiology.

[10] Cornell M, Kaderka R, Hild SJ, Ray XJ, Murphy JD, Atwood TF, Moore KL (2020). Noninferiority study of automated knowledge-based planning versus human-driven optimization across multiple disease sites. Int J Radiat Oncol Biol Phys.

[11] La Macchia M, Fellin F, Amichetti M (2012). Systematic evaluation of three different commercial software solutions for automatic segmentation for adaptive therapy in head-and-neck, prostate and pleural cancer. Radiat Oncol.

[12] Chen W, Wang C, Zhan W (2021). A comparative study of auto-contouring softwares in delineation of organs at risk in lung cancer and rectal cancer. Sci Rep.

